# Epinecidin‐1 (an active marine antimicrobial peptide): Effects on the survival of inoculated *Escherichia Coli* O157:H7 And *Staphylococcus aureus* bacteria, antioxidant, and sensory attributes in raw milk

**DOI:** 10.1002/fsn3.3514

**Published:** 2023-06-22

**Authors:** Ziba Salimnejhad, Hassan Hassanzadazar, Majid Aminzare

**Affiliations:** ^1^ Department of Food Safety and Hygiene, School of Public Health Zanjan University of Medical Sciences Zanjan Iran

**Keywords:** antioxidant property, Epinecidin‐1, *Escherichia Coli* O157:H7, Milk, *Staphylococcus aureus*

## Abstract

This study aimed to evaluate the effects of Epinecidin‐1 (Epi‐1) on total viable count (TVC), total psychrotrophic count (TPC), sensory attributes, and the survival of *Escherichia Coli* O157:H7 and *Staphylococcus aureus* bacteria inoculated in pasteurized milk samples during cold storage (4°C). Four treatments of milk samples were prepared including milk samples containing three concentrations of Epi‐1 (0.0025, 0.005, and 0.01%) and control (without Epi‐1). The treated milk samples were evaluated in vitro (minimum inhibitory concentration, Minimum bactericidal concentration, disk diffusion test, DPPH, reducing power assays) and in vivo (TVC, TPC, sensory properties, and enumeration of inoculated *E. coli* and *S. aureus*) during 9 days at cold storage. The best antibacterial and antioxidant power of Epi‐1 was observed at a concentration of 0.01%. Based on the MICs and MBCs, the most susceptible and resistant bacteria to Epi‐1 were *B. cereus* and *S. aureus* strains, respectively. The DPPH scavenging potential of Epi‐1 was in the range of 77%–80%. Treated samples containing 0.01% Epi‐1 had the lowest TVC and TPC and reached 3.9 and 2.96 CFU log/mL at the end day of storage. A decrease of 6 and 1.4 logs CFU/g of *E. coli* O157:H7 and *S. aureus* was seen in all treatments containing Epi‐1, respectively, on the last day of storage period. There are no unpleasant sensory properties in treated samples with Epi‐1. Our results indicate that Epi‐1 has good potential as a bio‐preservative to prevent raw milk spoilage and reduction of milk‐borne pathogens.

## Graphical abstract

Antimicrobial, antiviral, antitumor, immunomodulatory, and wound‐healing effects of Epinecidin‐1 (an active marine antimicrobial peptide) were reported in several studies related to medicine. There are no studies on food preservation aspects. Epi‐1 showed strong antibacterial effects against foodborne pathogens. Good antimicrobial and antioxidant results and pleasant sensory properties were seen in raw milk containing Epi‐1 during cold storage.

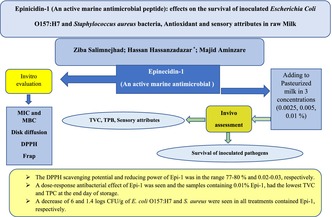

## 1 INTRODUCTION

Milk and dairy products are considered as great nutrient‐rich food in human diet. Their nutritional quality and shelf life are decreased by microbial and chemical spoilage. *Staphylococcus aureus*, *Escherichia coli*, *Salmonella strains*, *Listeria monocytogenes*, Mycobacterium strains, Campylobacter, Clostridia, and Brucella strains are common pathogens in milk and dairy products as a rich nutrient source with neutral pH and high‐water content (Alam et al., 2017). Raw milk is an important food for transmission of *E. coli* O157:H7 and other pathogens such as *S. aureus* (Dai et al., 2019; Garbaj et al., 2016). The young dairy cattle are a reservoir of *E. coli* O157:H7 (Garbaj et al., 2016). *S. aureus* is a pathogenic bacterium‐cause food poisoning primarily due to secretion of enterotoxins after contamination of milk and dairy products. Both bacteria can survive and grow in cold storage (4–10°C) (Hill & Kethireddipalli, 2013).

Food preservation techniques such as pasteurization, sterilization, and addition of preservatives as nonthermal technology help dairy industries to eliminate foodborne pathogens and extend the shelf life of milk and dairy products. This shelf life is affected by microbial load and contamination of food which is associated with quality deficiency of milk products (Ziyaina et al., 2018). The synthetic or natural preservatives are good candidates to control microbial growth and chemical spoilage and maintain the initial nutritional and sensory properties of food (Lotfi et al., 2018). Given the side effects of chemical preservatives, the preferences and tendency of consumers to use natural additives, antioxidant, and antimicrobial compounds has been increased (Pateiro et al., 2018). Natural preservatives derived from plants, animals, or microorganisms like essential oils; various plant extracts and antimicrobial compounds including bacteriocins, lactoperoxidase, lysozyme, and so on are the most populated compounds used in food industry (Patrzykat et al., 2002). To help reduce the possibility of milk and dairy products becoming contaminated with bacterial pathogens, natural antimicrobials could be added before or after processing as a bio‐preservative measure.

Antimicrobial peptides (AMPs) are known as potent antimicrobial derivatives extracted often from living organisms with a wide range of activities such as bacteriocins, lysozyme, and lactoperoxidase (Patrzykat et al., 2002). It was demonstrated that marine AMPs have a good potential to be used in human and aquaculture as a natural antibiotic against a plethora of marine microbes. The main attributes of AMPs are targeting the cell membrane and inner components of the pathogens based on the short chain (less than 50 amino acids), cationic, or amphipathic construction of them resulting in rapid killing of the pathogens (Chee et al., 2019; Narayana et al., 2015; Neshani et al., 2019). Among 3065 known AMPs, 125 marine AMPs are detected as novel antimicrobial agents and first line of defense against different pathogens in the sea that derived from fish and marine organisms such as cathelicidins, defensins, hepcidins, pleurocidin, moronecidin, dicentracin, Epinecidin‐1, chrysophsin, myxindin, misgurin, and gaduscidin (Huang et al., 2018; Pan et al., 2018).

Epinecidin‐1 (Epi‐1) is a linear cationic α‐helical peptide with 67 amino acids (an arranged sequence as H‐Gly‐Trp‐Gly‐Ser‐Phe‐Phe‐Lys‐Lys‐Ala‐Ala‐His‐Val‐Gly‐Lys‐His‐Val‐Gly‐Lys‐Ala‐Ala‐Leu‐Thr‐His‐Tyr‐Leu‐OH) belongs to piscidin family and its first isolation was from orange‐spotted grouper (*Epinephelus coioides*) (Chee et al., 2019; Huang et al., 2018; Pan et al., 2018; Raju et al., 2021). Antimicrobial, antiviral, antitumor, immunomodulatory, and wound‐healing effects of Epi‐1 were reported in previous studies related to medicine. Studies showed pore formation ability of Epi‐1 in membranes of bacteria resulted in lysis and their subsequent death which means it may be effective against other pathogens (Chee et al., 2019; Huang et al., 2018; Pan et al., 2010, 2018; Raju et al., 2021). Despite many studies devoted to study the antimicrobial properties of Epi‐1, no studies have been performed on the preservative effects of Epi‐1 in the food model, especially milk and dairy products. Hence, the aim of this study was to determine the in vitro antimicrobial and antioxidant effects of Epi‐1 as well as its effect on total count, sensory attributes, and the fate of inoculated *Escherichia coli* O157:H7 and *Staphylococcus aureus* in raw milk during refrigeration (4–8°C).

## 2 MATERIALS AND METHODS

### 2.1 Materials

Epinicide‐1, 2, 2‐diphenyl‐2‐picrylhydrazyl (DPPH) and other chemicals were purchased from Sigma‐Aldrich (Sigma Aldrich). All used culture media were purchased from Merck (Merck KGaA). Bacterial strains including *Escherichia coli* O157:H7 (ATCC 70728), *Staphylococcus aureus* (ATCC 25923, ATCC 29213, ATCC 9144), *Listeria monocytogenes* (ATCC 13932, ATCC 19114, ATCC 19115), *Escherichia coli* (ATCC 25922, ATCC 15224, ATCC 10536), *Pseudomonas aeruginosa* (ATCC 25619, ATCC 27853, ATCC 15442), *Bacillus cereus* (ATCC 11778, PTCC1665, PTCC 1857), *Salmonella enterica* subsp. *enterica* (ATCC 13076, ATCC 14028), and Shewanella SP (ATCC 1711) were provided lyophilized by Iranian Research Organization for Science and Technology (IROST).

### 2.2 Preparation of bacterial strains

In the beginning, a culture suspension of mentioned strains of each foodborne pathogen (*S. aureus*, *E. coli* O157: H7, *S. enteritidis*, *E. coli*, *B. cereus*, *P. aeruginosa*, and *S. putrefaciens*) was prepared based on the instruction of IROST manual for each bacterium. Then, the cocktail suspensions of the same species of pathogens were prepared by mixing equal volumes of each strain. A loopful of bacterial suspensions was cultured in brain–heart infusion (BHI) broth (Merck) to refresh bacterial strains and incubated at 37°C for 18–24 h. The turbidity created by bacterial growth was then adjusted with a standard McFarland 0.5 test and confirmed spectrophotometrically (Milton Roy Company) at a 600 nm wavelength and 0.08–0.1 absorbance to obtain 10^8^ CFU/mL bacterial count. A tenfold serial dilution in 0.1% peptone water was prepared for each cocktail of tested pathogens. A surface spreading count on plate count agar (PCA) at 37°C for 24 h was performed to confirm obtained enumerations (Hashemi et al., 2015).

Biological and antibacterial effects of Epi‐1 were evaluated using in vitro (determination of minimum inhibitory concentration [MIC], disk diffusion method, DPPH scavenging, and reducing power assays) and in vivo techniques (total count assay, enumeration of total psychrotrophic bacteria, inoculated bacteria, and assessment of sensory attributes), respectively.

### 2.3 In vitro assays

#### 2.3.1 Determination of minimum inhibitory concentration (MICs)

At first, Epi‐1 solutions were prepared in the range of 0.01, 0.005, 0.0025, 0.00125, and 0.000625 0.000312%. A standardized dilution (10^6^ CFU/mL) of each bacterium was prepared from stock solution. The MIC was determined with broth micro‐dilution method in 96‐well microplates. Briefly, 160‐μL BHI broth, 20 μL of bacteria (10^6^ CFU/mL), and 20 μL of different concentrations of Epi‐1 solutions (totally 200 μL) were mixed in the wells of microplates. Then, microplates were incubated at 37°C for 24 h. Chloramphenicol antibiotic solution and a mixture of BHI broth containing Epi‐1 without bacterial suspension were set as positive and negative control groups, respectively. The lowest concentration of the assayed antimicrobial agent (Epi‐1) that inhibits the visible growth of the bacterium is defined as minimal inhibitory concentration (MIC). The MIC dilution and two more concentrated test dilutions are plated and enumerated to determine viable population of bacteria. The lowest concentration at which an antimicrobial agent will kill 99.90% of microorganisms defined as minimum bactericidal concentration (MBC) (Hashemi et al., 2015).

#### 2.3.2 Disk diffusion method

The agar disk diffusion assay was used to determine the antimicrobial activity of the Epi‐1 based on the described method by Fadil et al. (2018) with some modifications. At first, 1 mL of each bacterial suspension (10^8^ CFU/mL) was spread on Muller Hinton Agar (MHA) plates and incubated for 20 min. Filter paper disks (6 mm in diameter) were impregnated by 10 μL of different dilutions of the Epi‐1 (0.01, 0.005, 0.0025, 0.00125, 0.000625, and 0.000312%). In addition, chloramphenicol disk (30 μg) was used as positive control to determine the sensitivity of the tested strains. These Petri dishes were incubated at 37°C for 24 h. All tests were performed in triplicate. The growth inhibition zone (GIZ) diameters were measured in millimeters (Fadil et al., 2018).

#### 2.3.3 2, 2′‐ diphenyl‐1‐picrylhydrazyl radical scavenging assay

The antioxidant capacity or radical scavenging potential of Epi‐1 solution was evaluated according to the described method by Fathollahi et al. (2019) with some modifications. Briefly, different concentrations (0.01, 0.005, and 0.0025%) of Epi‐1 and various concentrations of BHT (1 %) were prepared in methanol solvent. Then, 1 mL of DPPH solution (1 mM) prepared in methanol was added to 3 mL of the solutions and the mixture was mixed. Test tubes were placed for 30 min in a dark room. After this time, absorption rate was read at 517 nm with a spectrophotometer (Novaspec II; Pharmacia LKB). In the control sample, the active solution was replaced with 3‐mL methanol. Finally, the inhibition rate of DPPH radicals was determined using the following formula (Fathollahi et al., 2019).

$$\text{Free radical inhibition rate}\;\left(\%\right)=\left(\mathrm{AC}-\mathrm{AS}\right)/\mathrm{AC}\times 100\%$$

where AC and AS are the absorbance of the control and the sample, respectively.

#### 2.3.4 Reducing power assay.

The reducing power or electron‐donating capacity of different concentrations of Epi‐1 solution (0.01, 0.005, and 0.0025%) and BHT (1 %) was determined according to the described method by Canabady‐Rochelle et al. (2015) with some modifications. One milliliter of test solution was mixed with 2.5‐mL phosphate buffer (M = 0.2, pH = 6.0) and 2.5‐mL potassium ferricyanide (10 g/L) and placed in water bath (50°C) for half an hour. Then, after the addition of 2.5 mL of trichloroacetic acid 10% (W/V) to the samples, test tubes were centrifuged for 10 min at 1650 rpm speed. In the end, 2.5 mL of the supernatant of each sample was mixed with 2.5 mL DW and 0.5‐mL ferric chloride (1 g/L), and the absorbance of the Epi‐1 and blank tubes was measured spectrophotometrically (Milton Roy Company) at 700 nm after 10 min. High absorbance showed higher reducing power of the samples (Canabady‐Rochelle et al., 2015).

### 2.4 In vivo assays

#### 2.4.1 Preparation of pasteurized milk treatments

At first, some industrial pasteurized milk was purchased. Milk samples were boiled again for 1 min to ensure no contamination. Milk samples with pH = 6.5–7 were cooled for 3 h at 20°C in sterile conditions (Ebrahimiasl et al., 2018). Six treatments were prepared including: 1. Control sample (pasteurized milk sample without antimicrobial agent), 2–4. Pasteurized milk samples containing three concentrations of Epi‐1 (0.0025, 0.005, and 0.01%), 5. Pasteurized milk containing three concentrations of Epi‐1 (0.0025, 0.005, and 0.01%) inoculated with the cocktail of *S. aureus* strains (10^6^ CFU/mL), and 6. Pasteurized milk containing three concentrations of Epi‐1 (0.0025, 0.005, and 0.01%) inoculated with the *E. coli* O157:H7 (10^4^ CFU/mL).

#### 2.4.2 Total viable count

Total bacterial count of control sample and milk samples containing three concentrations of Epi‐1 were determined using the standard plate count method (SPC) during 9 days of cold storage with 3‐day intervals (28, 29). Briefly, 1 mL of the milk samples was transferred to tubes containing 9‐mL sterile 0.1% peptone water (PW) (Merck KGaA) to prepare a decimal dilution. A serial dilution was prepared in order to achieve a colony count of between 30 and 300 colonies per plate after spreading 1 mL of each dilution in plate count agar (PCA) (Merck KGaA) medium. All cultured agar plates were incubated at 37°C for 24–48 h (Bazargani et al., 2015).

#### 2.4.3 Total psychrotrophic count

Total count of psychrotrophic bacteria was enumerated using the SPC technique and incubated cultured plates for 10 days at 7°C (Mansour et al., 2021).

#### 2.4.4 Enumeration of inoculated pathogens in milk samples

To enumerate the inoculated bacteria, 1 mL of different inoculated milk samples was transferred to the tubes containing 9 mL of 0.1% PW and serial dilution was prepared in order to achieve a colony count of between 30 and 300 colonies per plate after spreading of 1 mL of each dilution in selective agar media for *S. aureus* (Mannitol salt agar) and *E. coli* O157:H7 (eosin methylene blue). All cultured agar plates were incubated at 37°C for 24–48 h (Abdalbeygi et al., 2022; Bazargani et al., 2015). The results were expressed in the form of the log CFU/mL.

### 2.5 Assessment of sensory attributes

In this study, assessment of sensory attributes was conducted in milk samples containing antimicrobial agent without pathogens. The sensory characteristics of different treatments included (color, odor, taste, and overall acceptability) were evaluated during 9 days of storage period with the 9‐point hedonic scoring scale test by 30 trained individuals. In this scaling method, the 1–9 scores were as 1 (intolerable), 2 (very bad), 3 (bad), 4 (tolerable), 5 (moderate), 6 (good), 7 (very good), 8 (super), and the score 9 is a highly acceptable scale (Excellent) for the samples (Bazargani et al., 2015).

### 2.6 Statistical analysis

All tests were performed in triplicate. Statistical data analysis was carried out using SPSS software ver. 25 (SPSS, Inc.). Data were expressed as mean ± standard deviation. Tukey's test was used to determine the significant difference among samples. Sensory data were analyzed using the Friedman nonparametric test. Statistical significance was stated at *p* < .05.

## 3 RESULTS AND DISCUSSION

### 3.1 In vitro antimicrobial activity of Epi‐1

The possibility of using antimicrobial peptides against pathogens was investigated previously. The results showed that antimicrobial peptides are safe and can be used for different purposes such as food preservative (Singh & Abraham, 2014).

The growth inhibition ability of different concentrations of Epi‐1 against some foodborne pathogens is demonstrated in Tables 1 and 2. According to the obtained results based on the MICs and MBCs, the most susceptible and resistant bacteria to Epi‐1 were *B. cereus* and *S. aureus* strains, respectively (Table 1). Growth inhibition of some human pathogens by Epi‐1 based on MIC has been reported in the range 12.5–50, 3–10, and 50 μg/mL by Chee et al. (2019) against *S. aureus*, *P. aeruginosa*, and *L. monocytogenes* strains, respectively, which are inconsistent in some cases with the results of this study. The reason for these differences in MIC could be due to differences in the subspecies of the studied bacteria (Chee et al., 2019; Huang et al., 2018; Pan et al., 2018). Compared to niacin and lysozyme as antimicrobial peptides against *S. aureus*, *P. aeruginosa*, *S. enteritidis*, and *E. coli* strains, the acceptable results were seen for Epi‐1 at neutral pH (6.5–7) reported by Moshtaghi et al. (2018).

**TABLE 1:** MIC and MBC assay (mg/mL) of Epi‐1 (%) against some foodborne pathogens.

	*E. coli*	*S. aureus*	*L. Monocytogenes*	*S. Enteritidis*	*P. aeruginosa*	*E. coli* O157: H7	*B. cereus*
MIC	0.0025	0.0025	0.0025	0.00125	0.0025	0.0025	0.000625
MBC	0.005	0.02	0.01	0.01	0.005	0.01	0.0025

**TABLE 2:** Growth inhibition zone (mm) of different concentrations (%) of Epi‐1 against some foodborne pathogens using disk diffusion method (Mean ± SD).

Foodborne pathogen	Epi‐1 concentrations (%)						Control
	0.01	0.005	0.0025	0.00125	0.000625	0.000312	
*L. monocytogenes*	13.33 ± 0.6^a^	11.33 ± 0.6^b^	11.33 ± 1.5^b^	7.33 ± 0.6^c^	0	0	20.16 ± 1.6
*S. aureus*	11.33 ± 1.1^a^	7.66 ± 0.6^b^	0	0	0	0	20.3 ± 1.6
*E. coli O157: H7*	14.66 ± 1.1^a^	13.33 ± 0.6^b^	11.33 ± 0.6^c^	9.66 ± 0.6^d^	0	0	25.3 ± 0.44
*P. aeruginosa*	11 ± 0^a^	8.33 ± 0.6^b^	6.66 ± 0.6^c^	0	0	0	23.5 ± 1.2
*E. coli*	14.33 ± 0.6^a^	12.33 ± 0.6^b^	11.33 ± 1.5^c^	9 ± 1^d^	0	0	23.2 ± 0.7
*B*. cereus	13.33 ± 0.6^a^	10.33 ± 0.6^b^	9 ± 1^c^	0	0	0	24.2 ± 0.75
*S. enteritidis*	12 ± 0^a^	10.66 ± 0.6^b^	7.33 ± 0.6^c^	0	0	0	24.3 ± 1.03

*Note*: Nonsimilar small letters in each row show significant difference among the Epi‐1 concentrations.

Abbreviation: SD, Standard deviation.

The growth inhibition zone (GIZ) based on disk diffusion increased with the increasing of Epi‐1 concentration and a dose–response was seen in the results (Table 2). The highest GIZs were related to control and 0.1 mg/mL concentration of Epi‐1 for all studied bacteria. The lowest and highest GIZs in this concentration belonged to *P. aeruginosa* and *E. coli* O157: H7 strains. Also, the highest resistance to Epi‐1 was related to *S. aureus* strains. There was a significant difference between different concentrations of Epi‐1 against each bacterium. The difference in sugars, linkages, and arrangement possibilities in the cell wall and LPS structure of studied bacteria is the probable reason for observed differences in MICs and GIZs between studied gram‐negative and gram‐positive bacteria in this study which may affect the efficacy of Epi‐1 against studied pathogens (Biernbaum et al., 2021).

### 3.2 In vitro antioxidant activity of Epi‐1

Antioxidant capacity of Epi‐1 to scavenge DPPH radicals and reducing potential of ferric ions are shown in Table 3. DPPH free radicals scavenging assay is one of the accepted mechanism for screening the antioxidant ability of biological materials. Ferric reducing power assay (FRAP) shows also the antioxidant capacity by the reduction of ferric iron (Fe3+) to ferrous iron (Fe2+) of antioxidants. As is shown in Table 3, compared to BHT (1%), Epi‐1 showed acceptable antioxidant capacity based on both assays. There were no significant differences among the used concentrations of Epi‐1. Recently, certain peptides with antioxidant activity have been isolated from proteins. The relationship between the presence of aromatic amino acids and the antioxidant properties of peptides has been reported by Shahidi and Zhong (2010). They reported that peptide fractions with high levels of histidine and hydrophobic amino acids showed higher adsorption capacity of DPPH (Shahidi & Zhong, 2010). Accordingly, a brief look at the amino acid sequence in the structure of Epi‐1 justifies its antioxidant effect (Chee et al., 2019).

**TABLE 3:** DPPH radical scavenging and reducing power capacity of Epi‐1 (Mean ± SD).

Epi‐1 (%)	DPPH scavenging assay	Reducing power assay
0.0025	79.94 ± 1.40^a^	0.02 ± 0.004^a^
0.005	79.26 ± 1.83^a^	0.02 ± 0.003^a^
0.01	77.07 ± 2.32^a^	0.03 ± 0.005^a^
BHT (1%)	88.63 ± 0.08^c^	0.03 ± 0.002^a^

*Note*: Nonsimilar small letters in each column show significant difference among the Epi‐1 concentrations.

Abbreviation: SD, Standard deviation.

### 3.3 In vivo antimicrobial effects of Epi‐1 (food model)

In this study, Epi‐1in three concentrations of 0.0025, 0.005, and 0.01% was added to the pasteurized milk samples as food model. Then, the total viable count (TVC), total psychrotrophic bacteria (TPB), sensory attributes, and the persistence of *E. coli* O157: H7 and *S. aureus* inoculated in milk samples were evaluated during refrigeration at the intervals of 0, 3, 6, and 9 days.

**FIGURE 1:**
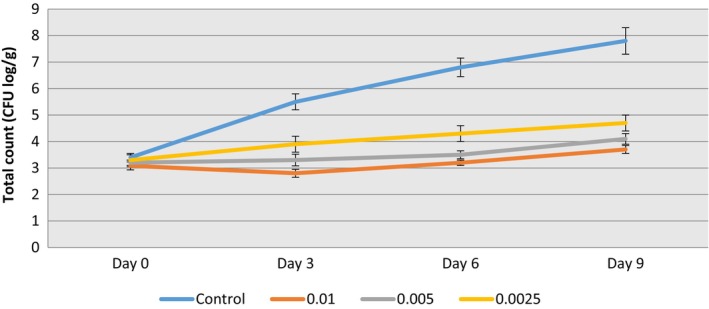
Total count enumerations of milk samples containing different concentrations of Epi‐1 (%) during 9‐day cold storage.

As shown in Figure 1, there is no significant difference in TVC (between the treatments 3.08–3.4 CFU log/mL) on the first day of storage (*p* > .05). The average TVC was higher significantly in the control samples than in other treatments on the third day (5.5 CFU log/mL) which reached 7.8 CFU log/mL on the last day of cold storage (*p* < .05). Treated samples containing 0.01% Epi‐1 had the lowest TVC on the ninth day of storage and reached 3.9 CFU log/mL after 9 days (*p* < .05). The TVC was constant in each treated milk sample containing Epi‐1 during storage period and the presence of Epi‐1 in milk was able to keep the total microbial count in milk at a constant and significant level for 9 days compared to the control sample.

The enumeration of TPB is shown in Table 4. The TPB count in pasteurized milk samples was 2.46–2.72 CFU log/mL on the first day of storage which increase significantly in control samples during the storage period (*p* < .05). Epi‐1 was able to inhibit the growth of TPB significantly and did not allow them to increase (*p* < .05). Producing high‐quality milk and dairy products is the aim of dairy industry throughout the world. However, TVB, TPB, and lactic acid bacteria count are the indicators of raw milk quality. Milk refrigeration is globally used to control mesophilic and thermophilic bacteria in raw milk, but this condition is ideal for the growth of psychrotrophic bacteria specially thermoduric species of gram‐positive and gram‐negative bacteria found in pasteurized milk including the genera bacillus, lactobacilli, lactococci and micrococci, arthrobacter, microbacterium, enterococcus, and corynebacterium which can spoil milk and have negative impact on the quality of dairy products (Suresh et al., 2019). The results of the present study showed that Epi‐1 was successful to control the growth of total mesophilic and psychrotrophic bacteria in the pasteurized milk samples at a dose–response reaction during refrigeration with a growth control potential in the range of 2.5–3.5 CFU log/mL during cold storage (Tables 3 and 4). The acceptable results were seen for Epi‐1 compared to the recognized antimicrobial peptides such as reuterin, nisin, and pediocin with a reduction ability in the range of 1–1.5 CFU log/mL of total count in the raw milk (Kumar et al., 2020).

**TABLE 4:** Total psychrotrophic bacteria count of milk samples containing different concentrations of Epi‐1 (%) during 9‐day cold storage.

	Storage days			
Epi‐1 concentrations	0	3	6	9
0.01	2.46 ± 0.05 ^Aa^	2.24 ± 0.02 ^Aa^	2.56 ± 0.1 ^Aa^	2.96 ± 0.01 ^Aa^
0.05	2.56 ± 0.1 ^Aa^	2.64 ± 0.15 ^Aa^	2.8 ± 0.15 ^Aab^	3.28 ± 0.3 ^Ab^
0.0025	2.64 ± 0.06 ^Aa^	3.12 ± 0.2 ^Aa^	3.44 ± 0.6 ^Bab^	3.76 ± 0.2 ^Bab^
Control	2.72 ± 0.25 ^Aa^	4.4 ± 0.1 ^Bb^	5.44 ± 0.2 ^Cc^	6.24 ± 0.1 ^Cd^

*Note*: Nonsimilar small letters in each raw show significant difference among the Epi‐1 concentrations. Nonsimilar capital letters in each column show significant difference among the Epi‐1 concentrations.

**FIGURE 2:**
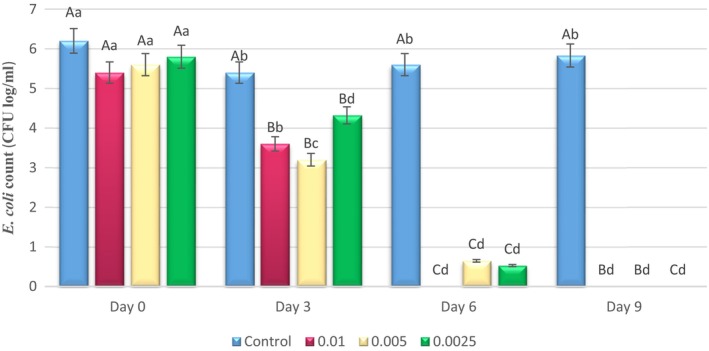
Changes in the *Escherichia. coli* O157:H7 count (log10 CFU/mL) inoculated in pasteurized milk samples containing different concentrations of EPI‐1 (0.01, 0.005, and 0.0025%) during 9 days of refrigeration. Different capital letters indicate a significant difference in each treatment among interval days (*p* < .05). Different small letters indicate a significant difference between treated samples on each day (*p* < .05).

**FIGURE 3:**
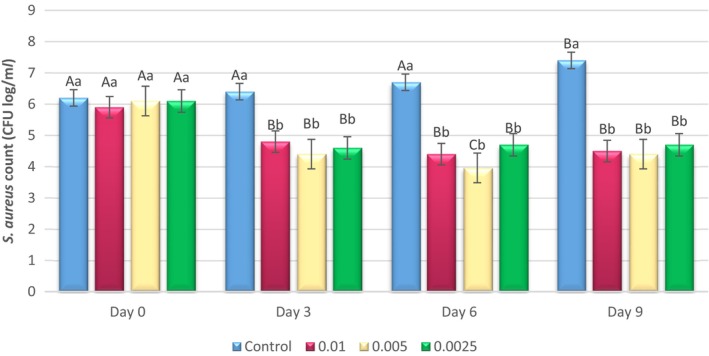
Changes in the *Staphylococcus. aureus* count (log10 CFU/mL) inoculated in pasteurized milk samples containing different concentrations of Epi‐1 (0.01, 0.005, and 0.0025%) during 9 days of refrigeration. Different capital letters indicate a significant difference in each treatment among interval days (*p* < .05). Different small letters indicate a significant difference between treated samples on each day (*p* < .05).

The load changes of inoculated *E. coli* O157:H7 and *S. aureus* (log CFU/mL) in pasteurized milk samples containing different concentrations of Epi‐1 during 9 days of refrigeration are shown in Figures 2 and 3, respectively. The results showed significant decrease of the *E. coli* population during storage period (*p* < .05) (Figure 2). The highest count of microbial load was related to the control treatment. On the ninth day of cold storage, all treatments containing Epi‐1 were free of any bacterial load except the control samples. The highest antibacterial effect was seen in treatments containing 0.01% Epi‐1, which showed a significant difference with other treatments (*p* < .05). In this study, the bacterial count of *E. coli* O157: H7 decreased in control samples over time compared to the first day. Tebyanian and Yousefi Sabete (2015) reported that the bacteria were affected by stress factors such as pH and reactive oxygen species (ROS). The shape of the bacteria tended to change to cocci or coccobacilli that lead to being able to survive under stressful conditions. It can be the main reason of declining of *E. coli* population in control samples. Compared to the activity of other recognized antimicrobial peptides such as enterocin KP and lactococcin BZ against *E. coli* O157: H7, Epi‐1 had an acceptable antipathogenic effect (Oncul & Yıldırım, 2019).

During the storage period, the number of *S. aureus* decreased in three concentrations of 0.01, 0.005, and 0.0025% until the third day, but remained almost constant on the sixth and ninth days. A significant difference was observed on each day compared to the control sample (*p* < .05). There was no significant difference among the different concentrations in treated samples during storage period. At the end of the storage period, the highest amount of bacterial load was related to the control treatment (7.4 CFU log/mL). A logarithmic reduction (1.5 log CFU/mL) of *S. aureus* was seen in treatments containing Epi‐1 on the ninth day of storage period.

Epi‐1 had a greater antibacterial effect on *E. coli* O157: H7 than *S. aureus*. Differences in the membrane structure of gram‐negative bacteria compared to gram‐positive bacteria and the tendency of the cationic structure of Epi‐1 to bind with the lipopolysaccharide membrane of the gram‐negative bacterial membrane may be the main reason for the different antibacterial effect of Epi‐1 (Raju et al., 2021).

An important question in this study was that if Epi‐1 was persistent during storage period? According to previous studies, antimicrobial effects of antimicrobial peptides depend on the physical and chemical structure of food such as fat content, pH, and temperature, and preservation techniques such as gamma irradiation (Huang et al., 2017). Huang et al. (2017) showed that high antimicrobial activity of Epi‐1 was seen under low pH conditions and high temperature or gamma ray (25 KGY) can reduce the antimicrobial activity of Epi‐1. In this study, the pH measurement on the ninth day in three concentrations showed that the acidity of milk was almost constant (pH = 6.3). It was reported that phospholipids in milk fat can reduce available AMPs such as nisin by binding to a large portion of the protein to bind with membrane of bacterial cells (Khelissa et al., 2020). The obtained results showed that Epi‐1 was able to control the TVC and TPB during the storage period and considering the reductions 6 and 1.5 logarithmic of *E. coli* O157: H7 and *S. aureus* on the ninth day in all treatments containing Epi‐1, respectively; it can be concluded that Epi‐1 persisted in milk samples for 9 days during the experimental period.

**FIGURE 4:**
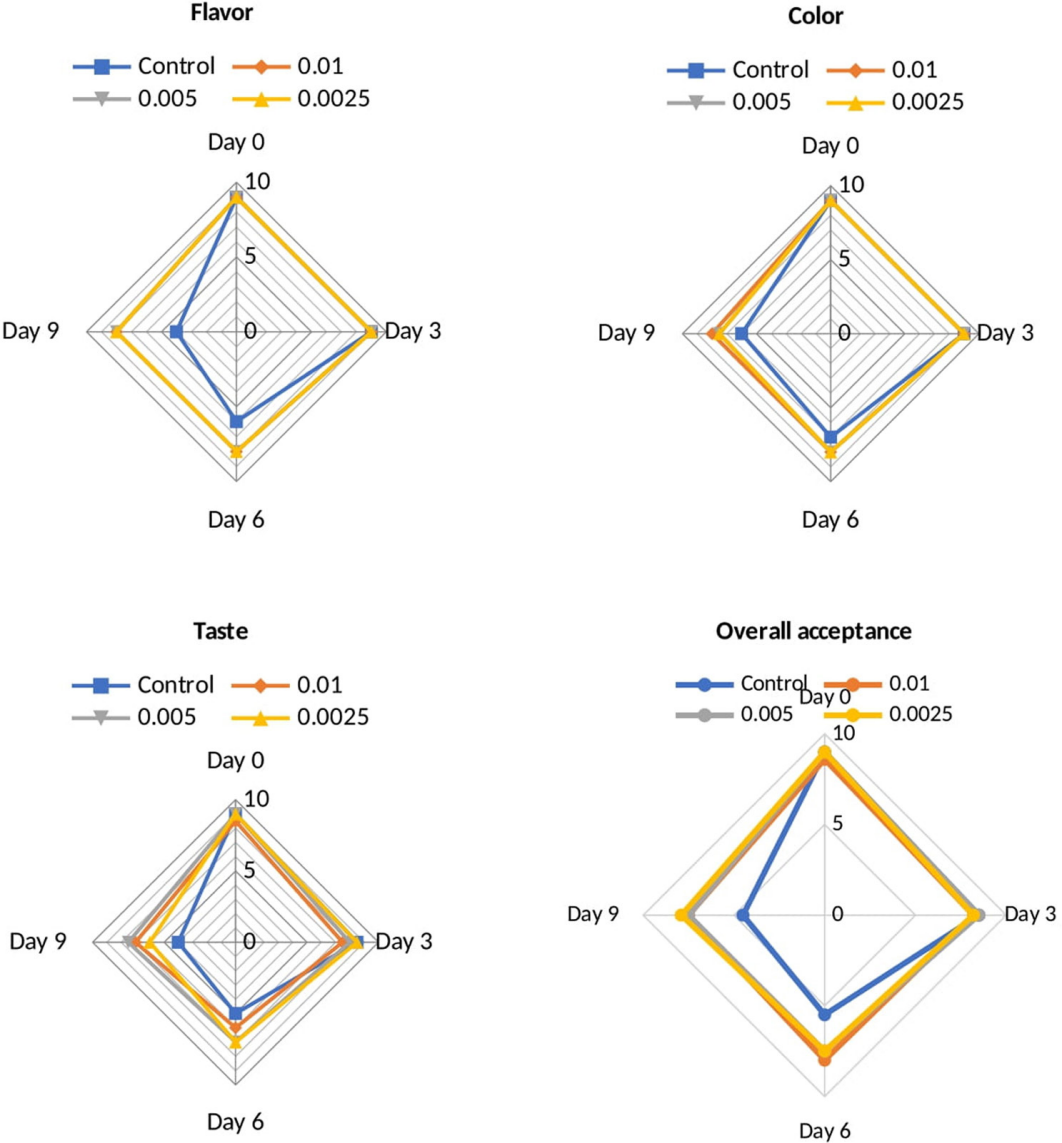
Sensory attributes of milk samples treated with different concentrations of EPI‐1 (0.01, 0.005, and 0.0025%) during 9‐day storage in the refrigerator.

The results of sensory evaluation are shown in Figure 4. There was no significant difference in terms of the items of sensory attributes among the treated and control samples until third day of storage (*p* > .05). The taste, color, and overall acceptance of the control samples were significantly different from the other treated samples after the third day of storage period (*p* < .05). The overall acceptance of all treated samples with different concentrations of Epi‐1 was higher than very good score with no significant difference among the Epi‐1 concentrations and no unpleasant sensory properties at the end of the storage period (*p* > .05).

## 4 CONCLUSION

Considering the results of this study, Epi‐1 has strong antibacterial effects against foodborne pathogens. The best antibacterial and antioxidant power of Epi‐1 was observed at a concentration of 0.01%. According to the MICs and MBCs, the most susceptible and resistant bacteria to Epi‐1 were *B. cereus* and *S. aureus* strains, respectively. The DPPH scavenging and RP potential of Epi‐1 were in the range of 77%–80% and 0.02–0.03. Treated samples containing 0.01% Epi‐1 had the lowest TVC and TPC and reached 3.9 and 2.96 CFU log/mL at the end day of storage. A decrease of 6 and 1.4 logs CFU/g of *E. coli* O157:H7 and *S. aureus* were seen in all treatments containing Epi‐1, respectively, on the last day of storage period. There are no unpleasant sensory properties in treated samples with Epi‐1 (*p* > .05). Given the good antimicrobial and antioxidant results of Epi‐1, it can be concluded that Epi‐1 can be used to extend the shelf life of raw milk and dairy products.

## AUTHOR CONTRIBUTIONS

**Ziba Salimnejhad:** Investigation (equal); methodology (equal); project administration (equal); resources (equal); writing – original draft (equal). **Hassan Hassanzadazar:** Conceptualization (lead); data curation (equal); formal analysis (lead); investigation (equal); methodology (equal); resources (equal); supervision (equal); writing – review and editing (equal). **Majid Aminzare:** Data curation (equal); methodology (equal); writing – review and editing (equal).

## CONFLICT OF INTEREST STATEMENT

The authors declare no conflicts of interest regarding this article.

## Data Availability

Data available on request from the authors (In persian).
